# Collecting Data To Assess SARS Interventions

**DOI:** 10.3201/eid1007.030749

**Published:** 2004-07

**Authors:** R. Douglas Scott, Edward Gregg, Martin I. Meltzer

**Affiliations:** * Centers for Disease Control and Prevention, Atlanta, Georgia, USA

**Keywords:** severe acute respiratory syndrome, program effectiveness, cost-effectiveness, dispatch

## Abstract

With cases of severe acute respiratory syndrome (SARS) occurring across geographic regions, data collection on the effectiveness of intervention strategies should be standardized to facilitate analysis. We propose a minimum dataset to capture data needed to examine the basic reproduction rate, case status and criteria, symptoms, and outcomes of SARS.

First detected in China, confirmed and probable cases of severe acute respiratory syndrome (SARS) have now appeared in at least 30 countries in five continents. SARS is the first new severe infectious disease to occur in the 21st century, and little is known about its epidemiologic features (1). To assess the effect of SARS on public health and outcomes, data are needed about who becomes ill, how they contracted their illness, and the sequelae.

A minimum set of data on intervention effectiveness should be collected in a uniform manner from each identified SARS case-patient at each location. Without such standardization, datasets from different locales may not be sufficiently comparable, thereby limiting the ability to scientifically evaluate both the effect of SARS and the interventions to control and prevent its spread.

We propose a minimum set of epidemiologic and clinical variables that should be among the top priorities when designing data collection protocols related to SARS interventions. We set priorities for the variables in the minimum dataset as a guide for agencies unable to collect all the recommended data. Additionally, we summarize the health measures constructed from each of the variables, along with the possible policy implications, to provide further guidance to health agencies regarding the importance of each variable. A case study is presented in Appendix.

Previous tools have been used to understand the spread of SARS and associated illnesses (2). These tools have not provided all necessary data to facilitate modeling usefulness and cost-effectiveness of interventions. Researchers have published results from relevant epidemiologic data, but no forms of itemized data are readily available (3). Our minimum dataset differs from minimum reporting requirements recently published by the World Health Organization (WHO) (2). WHO data templates include a daily summary of SARS cases to be reported at the national level and a case-reporting form that contains detailed clinical information (based on current WHO case definitions), including patient demographics, exposure, contact follow-up, daily reporting of symptoms, hospital admission, final case classification, and final case status. The dataset we propose captures information on length of exposure, incubation period from exposure to symptom onset, and use of health care resources (e.g., length of hospitalization, length of isolation, and admission to intensive care) not currently collected by WHO's template.

## Proposed Minimum Dataset and Data Prioritization

**Figure 1:**
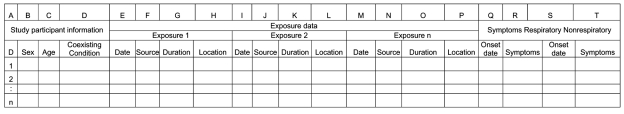
Schematic of table illustrating the epidemiologic data needed to evaluate impact of SARS and interventions: data relating to exposures and date of onset of symptoms. Data entry columns allow for multiple exposures and can expand as needed. Suggestions for coding the data for this table are given in Table A1. Note: to download this table for use, see PDF version.

**Figure 2:**
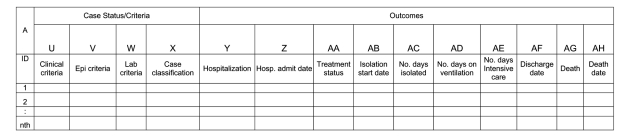
Schematic of table illustrating the epidemiologic data needed to evaluate impact of SARS and interventions: data relating to case status and outcomes. Suggestions for coding the data for this table are given in Table A2. Data entry columns move according to footnotes in Table A2 (letters at top of each column are used in describing data and rationale for each data point). Note: to download this table for use, see PDF version.

Figures 1 and 2 illustrate the minimum epidemiologic variables needed to evaluate the public health effect of SARS and the cost of interventions. These data would provide the evidence to determine key epidemiologic relationships, including the incubation period (time from exposure to onset of symptoms), the onset of symptoms leading to hospitalization, and the outcomes resulting from treatment (either discharge of patient or death). Descriptions of the variables listed in Figures 1 and 2, along with suggestions for coding, are provided in Tables A1 and A2. For all tables, the column heading corresponds with the variable name (e.g., A represents the case identification [ID] number, B represents sex, C represents age).

Figure 1 captures case-patient demographics, exposures, and symptoms. Suggested coding for demographic variables (Tables A1 and A2) include patient ID and age as continuous variables and sex and coexisting conditions (e.g., cardiovascular disease, diabetes) or syndromes (HIV/AIDS) as categorical variables. Other categories for coexisting conditions can be added as appropriate (e.g., smoking). An important distinction should be made between patients who have no known diagnosed coexisting conditions (coded as none known) as opposed to patients for whom information about coexisting conditions is not available or missing (coded as unknown).

In Figure 1, exposure variables and their suggested coding include date (DD/MM/YY), source (whether the source is already identified and included in the data table as an observed patient with an assigned ID or whether the source is unknown), duration of exposure (<30 minutes, 30–59 minutes, or >60 minutes), and locale (whether exposure occurred at home, in a hospital, or some other location). The same variables are measured for each exposure, and the table can be expanded to collect information on all known exposures.

Symptoms are categorized as either respiratory or nonrespiratory. For each symptom, onset date and type (a categorical variable that can be expanded for patients with multiple symptoms) are collected. Suggested categories for symptoms include fever, myalgia, dyspnea, headache, chills, diarrhea, nausea, sore throat, arthralgia, chest pain, productive cough, nonheadache neurologic symptoms (e.g., dizziness), rhinorrhea or runny nose, vomiting, and abdominal pain. The list of symptom categories can be revised or extended as needed.

Figure 2 contains information on case criteria, along with health outcomes associated with the case. Categorical variables making up case status include the clinical case criteria (either asymptomatic or mild respiratory illness, moderate illness, severe respiratory illness, or none), epidemiologic criteria (travel within 10 days to infected area, close contact, both, or none), laboratory confirmation (yes, no, or undetermined), and case classification (probable, suspected, or noncase).

Outcome variables include hospitalization (along with admission date if hospitalized), treatment status (antiviral agent, antibacterial agent, or other treatment), isolation start date, number of days isolated (a continuous variable), number of days on ventilation or in intensive care (continuous variables), discharge date (0 if still hospitalized), death (yes or no), and date of death. Appendix provides an example of Figures 1 and 2 filled out with data from four "typical" case-patients. The variable categories from Table A1 can be readily extended or revised as new information about SARS becomes available. The footnotes offer the definitions that served as the basis for the suggested categories.

## Priority Classification Groups

Tables A1 and A2 also provide proposed priority classification groups for each variable listed in Figures 1 and 2. Variables that are labeled "priority group 1" represent the most important set of variables, and those labeled as "priority group 3," the least important. The Table provides a summary of how each variable contributes to important health policy questions related to the SARS outbreak. Taken together, these tables can provide guidance to health organizations regarding which data should be collected so that the needed policy analysis can be conducted.

Priority group 1 variables (sex, age, date and source of exposure, date of symptom onset, and case status and criteria variables) contain the information on the transmission rate of the disease and incubation periods. These variables provide crucial information in determining the basic reproduction number of an infection (defined as the expected number of secondary infectious cases resulting from one primary case in a susceptible population) (4,5). This measure is vital for estimating the impact of control measures to reduce the transmission of SARS (4,5). Priority group 2 variables (duration and locale of exposure; hospitalization, including start date; isolation, including start date; and death, including date of death) provide information that can be used to evaluate the risk for hospitalization or death associated with exposure, length of incubation, and impact of isolation. Priority group 3 variables (coexisting conditions; categories of symptoms; treatment status; ventilation or intensive care, including start date; and date of discharge) are not essential information for containing SARS outbreaks but provide additional information about healthcare resources (treatment and intensive care) used to treat SARS patients. Priority group 3 variables can also be used by hospital administrators and public health officials to plan and prepare for a sudden change in resource use during a catastrophic infectious disease outbreak (e.g., pandemic influenza) (6).

## Conclusions

The emergence of a novel disease like SARS, which requires a global public health response to contain its spread, has illustrated the need for collecting effectiveness data in a uniform manner. Given the potential for a large variation in location-specific circumstances, producing a single questionnaire that would be entirely suitable for all locales would be difficult. Figures 1 and 2 illustrate some of the most important data needed to understand and control the disease. The tables present a standardized protocol and approach for ensuring that all the proposed data have been collected. As an illustration of the use of the tables, a case study is presented in Tables A3 and A4. Identifying effective interventions during an outbreak becomes important in managing public health resources. The minimum dataset proposed here provides a basis for standardizing the collection of data from various geographic locations, thereby facilitating the analysis of SARS interventions.

## Appendix

### Example Data (entries in Tables A1 and A2)

### Hypothetical Patient 1

A 55-year-old man traveled to mainland China from March 5 to March 15. By March 18, the man had a temperature of 38.5°C, cough, and sore throat. He was hospitalized on March 20, and results from radiographic tests indicated pneumonia. The patient was given a course of antimicrobial drugs, and the fever resolved. By March 23, the patient began complaining of chest pains and had difficulty breathing. At this point, SARS was suspected, and the patient was moved into isolation in an intensive care unit the same day and had to be intubated for 5 days. Results from the polymerase chain reaction (PCR) test indicated the presence of the SARS-associated coronavirus. The patient began antiviral treatment and remained isolated in intensive care until April 14. Infection resolved, and the patient was discharged on April 16. The patient had no previous history of serious respiratory illness or any other serious coexisting conditions, and the source of the exposure could not be determined.

### Hypothetical Patient 2

The 50-year-old wife of patient 1 was exposed to SARS upon her husband's return on March 15. On March 23, a cough developed, and she had shortness of breath and chills. She was hospitalized on March 24 with a temperature of 38.1°C. Radiographic tests indicated evidence of pneumonia. Because SARS was suspected, the patient was placed on antiviral treatment, and SARS was confirmed by PCR testing. Patient 2 was placed in isolation on a medical ward on March 25 and remained there until April 11. She was discharged on April 12. This patient was also diabetic.

### Hypothetical Patient 3

The 15-year-old daughter of patients 1 and 2 also became exposed to SARS when her father returned on March 15. She has a mild asthma condition. On March 23, she complained of stomach pain and, upon examination, had a temperature of 37.7°C. She was hospitalized on March 25 for observation, but no further symptoms developed. PCR testing returned negative results for the SARS coronavirus. Her fever resolved, and she was released on March 27.

### Hypothetical Patient 4

The 23-year-old daughter of patients 1 and 2, who lived away the family home, became exposed to SARS through contact with both patients 1 and 2 upon a brief visit (lasting <1 hour) on March 19. By March 27, she had a cough and a temperature of 38.1°C. She contacted her physician and was hospitalized on March 28 for observation and evaluation. Results of radiographic testing did not show pneumonia. She began an antiviral treatment program and began to improve. A PCR test was not completed because her fever resolved, and no other symptoms developed. She was discharged on April 1.

## Supplementary Material

Figure 1 Table PDF Empty table used in Figure 1.

Figure 2 Table PDF Empty table used in Figure 2.

## Appendix

**Table A1:** Explanation of data in Figure 1^a^

Column	Priority group	Variable	Type	Suggested coding	Comments/details
Participant information					
A	1	Patient ID	Numeric or character	1, 2, 3, …n	
B	1	Sex	Categories	1 = male 2 = female	
C	1	Age	Continuous	e.g. 25, 26	
D	3	Coexisting condition	Categories	0 = none known 1 = CVD 2 = diabetes 3 = COPD 4 = HIV/AIDS 5 = other 6 = unknown	Important to differentiate none known from unknown
Exposure data^a^					
Exposure 1					
E	1	Date	Date	DD/MM/YY	
F	1	Source ID	Numeric or character	ID of source patient or unknown	Identify, using patient ID, the presumed source of infection
G	2	Duration	Categories	1 = <30 min 2 = 30–59 min 3 = >60 min	Other categories of duration of exposure can be used. Must define "exposure"
H	2	Locale	Categories	1 = home 2 = hospital 3 = other	Other exposure locales can be added as needed
Exposure 2					
I–L and M–P	^b^	^c^	^c^	^c^	^c^
Symptoms, respiratory					
Q	1	Symptom onset date	Date	DD/MM/YY	Need to define the term "symptom"
R	3	Symptom category	Categories	^d^	Record all that are applicable and adjust as needed
Symptoms, non respiratory					
S	1	Symptom onset date	Date	DD/MM/YY	Need to define the term "symptom"
T	3	Symptom category	Categories	^d^	Record all that are applicable and adjust as needed

^a^Can expand to as many exposures as needed. ^b^Same as E–H. ^c^Repeat for each exposure. ^d^1 = fever; 2 = cough; 3 = myalgia; 4 = dyspnea; 5 = headache; 6 = malaise; 7 = chills; 8 = diarrhea; 9 = nausea/vomiting; 10 = sore throat; 11 = arthralgia; 12 = chest pain; 13 = productive cough; 14 = vomiting; 15 = rhinorrhea/runny nose; 16 = nonheadache neurologic symptoms (e.g., dizziness); 17 = abdominal pain.

**Table A2:** Explanation of data in Figure 2

Column	Priority group	Variable	Type	Suggested coding	Comments/details
Participant information					
A	1	Patient ID	Continuous	1, 2, 3, …n	
Case status/criteria					
U^b^	1	Clinical case criteria	Categories	1 = asymptomatic/mild respiratory illness; 2 = moderate illness; 3 = severe respiratory illness; 4 = none	See definitions in footnote^a^
V^c^	1	Epidemiologic criteria	Categories	1 = travel within 10 days to infected area; 2 = close contact; 3 = both; 4=none	Record all that apply See definitions in footnote^a^ Define close contact (e.g., within 1 min for X amount of time)
W^d^	1	Laboratory confirmed	Categories	1 = yes; 2 = no; 3 = undetermined	See definitions in footnote^a^
X^e^	1	Case classification	Categories	1 = probable; 2 = suspected; 3 = noncase	Classify using data from rows S, T, U
Outcome information					
Y	2	Hospitalization	Categories	1 = hospitalization; 2 = no hospitalization	
Z	2	Hospitalization date	Date	DD/MM/YY	
AA	3	Treatment status	Categories	1 = antiviral agent; 2 = antimicrobial agent; 3 = other (describe)	Suggestions/ recommendations? Particular interest is in nonhospitalized case-patients
AB	2	Isolation start date	Date	DD/MM/YY (or 0 = not in isolation)	Date on which patient was put into isolation for infection control purposes so as to prevent transmission
AC	2	No. of days isolated	Continuous	0,1,2,3,…..n	
AD	3	No. of days on ventilation	Continuous	0,1,2,3,…..n	
AE	3	No. of days in intensive care	Continuous	0,1,2,3,…..n	
AF	3	Discharge date	Date	Date; 0 if still in hospital	
AG	2	Death	Categories	1 = dead; 2 = alive	
AH	2	Date of death	Date	DD/MM/YY	

^a^All categorical definitions for coexisting conditions and symptoms are illustrative and do not imply any order or ranking. ^b^Clinical criteria - illustrative classification criteria: 1) Asymptomatic or mild respiratory illness; 2) Moderate respiratory illness: temperature of >100.4°F (>38°C), and >1 clinical findings of respiratory illness (e.g., cough, shortness of breath, difficulty breathing, or hypoxia); 3) Severe respiratory illness: temperature of >100.4°F (>38°C), and >1 clinical findings of respiratory illness (e.g., cough, shortness of breath, difficulty breathing, or hypoxia), and radiographic evidence of pneumonia, or respiratory distress syndrome, or autopsy findings consistent with pneumonia or respiratory distress syndrome without an identifiable cause. ^c^Epidemiologic criteria-illustrative classification criteria: travel (including transit in an airport) within 10 days of onset of symptoms to an area with current or recently documented or suspected community transmission of SARS; or close contact (e.g., having cared for or lived with a person known to have SARS or having a high likelihood of direct contact with respiratory secretions and/or body fluids of a patient known to have SARS, including close conversation [<3 feet]) within 10 days of onset of symptoms with a person known or suspected to have SARS infection. _d_Laboratory criteria-illustrative classification criteria: 1) Yes = confirmed: Detection of antibody to SARS-associated coronavirus (SARS-CoV) in a serum sample; or detection of SARS-CoV RNA by reverse transcriptase–polymerase chain reaction (RT-PCR) confirmed by a second PCR assay, by using a second aliquot of the specimen and a different set of PCR primers, or isolation of SARS-CoV; 2) No = negative: absence of antibody to SARS-CoV in convalescent-phase serum obtained >28 days after symptom onset; 3) Undetermined = laboratory testing either not performed or incomplete. eCase classification-illustrative classification: 1) Probable case: meets the clinical criteria for severe respiratory illness of unknown cause and epidemiologic criteria for exposure; laboratory criteria confirmed or undetermined; 2) Suspected case: meets the clinical criteria for moderate respiratory illness of unknown cause, and epidemiologic criteria for exposure; laboratory criteria confirmed or undetermined. The illustrative criteria and classifications used here are based on CDC Updated interim U.S. case definition for severe acute respiratory syndrome (SARS) (http://www.cdc.gov/ncidod/sars/casedefinition.htm).

**Table A3:** Schematic of table with the epidemiologic data to evaluate impact of SARS and interventions

Data entry columns (letters at top of each column are used in describing data and rationale for each data point)																			
A	B	C	D	E	F	G	H	I	J	K	L	M	N	O	P	Q	R	S	T
Subject Information				Exposure data												Symptoms			
				Exposure #1				Exposure #2				Exposure # 3				Respiratory		Nonrespiratory	
ID	Sex	Age	Coexisting conditions	Date	Source	Duration	Location	Date	Source	Duration	Location	Date	Source	Duration	Location	Onset date	Symptoms	Onset date	Symptoms
1	1	55	0	0	0	0	0	n/a				n/a				18/03/03	1,2,10		
2	2	50	2	15/03/03	1	3	1	n/a				n/a				23/03/03	2,3,4,7		
3	2	15	0	15/03/03	1	3	1	n/a				n/a				n/a	n/a	23/03/03	1,15
4	2	23	5	19/03/03	1	2	1	19/03/03	2	2	1	n/a				27/03/03	1,2		

**Table A4:** Schematic of table illustrating the epidemiologic data to evaluate impact of SARS and interventions: data relating to case status and outcomes

Data entry columns (letters at top of each column are used in describing data and rationale for each data point														
A	U	V	W	X	Y	Z	AA	AB	AC	AD	AE	AF	AG	AH
ID	Case status / criteria				Outcomes									
	Clinical Criteria	Epi criteria	Lab criteria	Case classification	Hosp.	Hosp admit date	Treatment status	Isolation start date	Number days isolated	Number days on ventilation	Number days intensive care	Discharge date	Death	Death date
1	3	1	1	1	1	20/03/03	1,2	23/03/03	23	5	23	16/04/03	2	n/a
2	3	2	1	1	1	24/03/03	2	25/03/03	19	0	0	12/04/03	2	n/a
3	4	2	2	3	1	25/03/03	2	0	0	0	0	25/03/03	2	n/a
4	2	2	1	2	1	26/03/03	2	0	0	0	0	01/04/03	2	n/a

**Table:** Potential calculations and policy implications from collected data

Variablesa	Who infected whom	Monitoring of disease spread and impact of interventions
E, I, M, and Q	Incubation period(s)	How soon should an exposed person be identified and placed in quarantine
A, B, C, F, J, and N	Who infected whom	Monitoring of disease spread and impact of interventions
E, I, M, G, K, O, Q, H, L, and P	When and where an infectious person infects another and duration of disease	Evaluation of infectiousness at different stages of disease and development or refinement of recommendations for persons exposed to SARS
D, E, I, M, G, K, O, H, L, P W, X, and AF	Effect of preexisting medical conditions on risk for hospitalization and death	Evaluation of medical response, with initial medical contact and treatment based on patients' risk factors
D, E, I, M, G, K, O, H, L, P, W, X, Y, Z, AA, AB, AC, AD, AE, and AF	Effect of certain preexisting conditions, type of contact, and length of incubation on increased risk for hospital isolation, ventilation, and intensive care	Evaluation of medical response, with analyses of how patients' risk factors impact allocation of hospital-based resources
R, S, T, U, V, and W	Classification of possible SARS cases	Evaluation of medical response, with degree of certainty of SARS diagnosis impacting allocation of health care resources
E, I, M, F, J, N, H, L, P, Q, W, Z, and X	Effect of isolation on spread of disease	Evaluation of interventions' effect on slowing and deterring the spread of disease
AG and AH	Death as an outcome	Evaluation of the severity of the outbreak

^a^From data entry columns, Figures 1 and 2.
